# Protocol for Urgent and Emergent Cases at a Large Academic Level 1 Trauma Center

**DOI:** 10.7759/cureus.3973

**Published:** 2019-01-28

**Authors:** Karim Ahmed, Corinna Zygourakis, Sammy Kalb, Zach Pennington, Camilo Molina, Terry Emerson, Nicholas Theodore

**Affiliations:** 1 Neurosurgery, Johns Hopkins Hospital, Baltimore, USA; 2 Neurosurgery, Barrow Neurological Institute, Phoenix, USA; 3 Surgery, Johns Hopkins Hospital, Baltimore, USA

**Keywords:** urgent surgery, emergency surgery, level 1 trauma center, trauma surgery, tertiary care center

## Abstract

Background

Level 1 trauma centers are capable of caring for every aspect of injury and contain 24-hour in-house coverage by general surgeons, with prompt availability of nearly all other disciplines upon request. Despite the wide variety of trauma, currently reported protocols often focus on a single surgical service and studies describing their implementation are lacking. The aim of the current study was to characterize all urgent and emergent cases at a large academic Level 1 trauma center, characterize the specialty and nature of emergent operative cases, and assess the efficacy of the institutional trauma protocol on timing of surgery.

Methods

For this retrospective review, all urgent and emergent cases treated at a single institution, during a 34-month period (January 1, 2015–October 31, 2017), were identified. All included cases were subject to the Institutional Guidelines for Operative Urgent/Emergent Cases. Demographic characteristics for non-elective surgical emergent cases were compiled by level of urgency and operating room (OR) waiting times were compared by year, department, and Level.

Results

A total of 11,206 urgent and emergent operative cases were included, among over 16 surgical departments. Level 2 cases represented the majority of urgent/emergent cases (33%–36%), followed by Level 3 (25%–26%), Level 1 (21%–22%), Level 4 (12%–16%), and Level 5 (2%–4%). Univariate analysis demonstrated that the proportion of urgent and emergent cases, by level of urgency, did not significantly differ between each year. Operating room waiting time decreased significantly over each year from 2015, 2016, and 2017: 193.40 ± 4.78, 177.20 ± 3.29, and 82.01 ± 2.98 minutes, respectively.

Conclusions

To the authors’ knowledge, this is the first study to characterize all urgent and emergent cases at a large academic Level 1 trauma center, outline the specialty and nature of emergent operative cases, and assess the efficacy of the institutional trauma protocol on surgical waiting times over a 34-month period.

## Introduction

The American Trauma Society and American College of Surgeons (ACS) designate a Level 1 trauma center as one capable of caring for every aspect of injury and containing 24-hour in-house coverage by general surgeons, with prompt availability of orthopedic surgery, neurosurgery, anesthesiology, emergency medicine, radiology, internal medicine, plastic surgery, oral and maxillofacial surgery, pediatric and critical care [1]. There is well-established literature, specific to various surgical specialties, describing the nature of cases classified as urgent/emergent, and the optimal timing for such cases [2-6]. Treatment delays for emergency surgery also significantly increase the economic impact of care, due largely to complications and length of hospital stay [7].

However, there is a lack of descriptive studies that outline all urgent and emergent cases seen at a Level 1 trauma center, identifying the most represented surgical departments and type of cases. Additionally, there is a lack of reported standardized triage protocols that take into consideration appropriate timing for such non-elective urgent cases. Current triage protocols that have been reported in the literature [8-10] underrepresent the variety of surgical specialties present in a Level 1 trauma center, and studies demonstrating their implementation and efficacy are limited. The aim of the current study was to summarize all urgent and emergent cases at a large academic Level 1 trauma center, characterize the specialty and nature of emergent operative cases, and assess the efficacy of the institutional trauma protocol on timing of surgery.

## Materials and methods

Institutional guidelines for operative urgent/emergent cases

In an effort to improve waiting times for urgent/emergent surgical cases, and ensure appropriate care for all patients, the Institute for Healthcare Optimization (IHO) was consulted to assist in designing a set of institutional guidelines for the triage of surgical cases. This group has previously applied variable methodology (VM), a concept of appropriating limited resources accounting for variability in healthcare delivery and acuity, successfully in numerous hospitals and institutions [5,11]. An Executive Steering Committee, Advisory Committee, and Working Committee – each consisting of clinicians, hospital administrators, and consultants – were assembled. In combination with variable methodology, queuing theory is an accurate tool to determine the expected supply of a hospital resource and its allocation [12].

**Figure 1:**
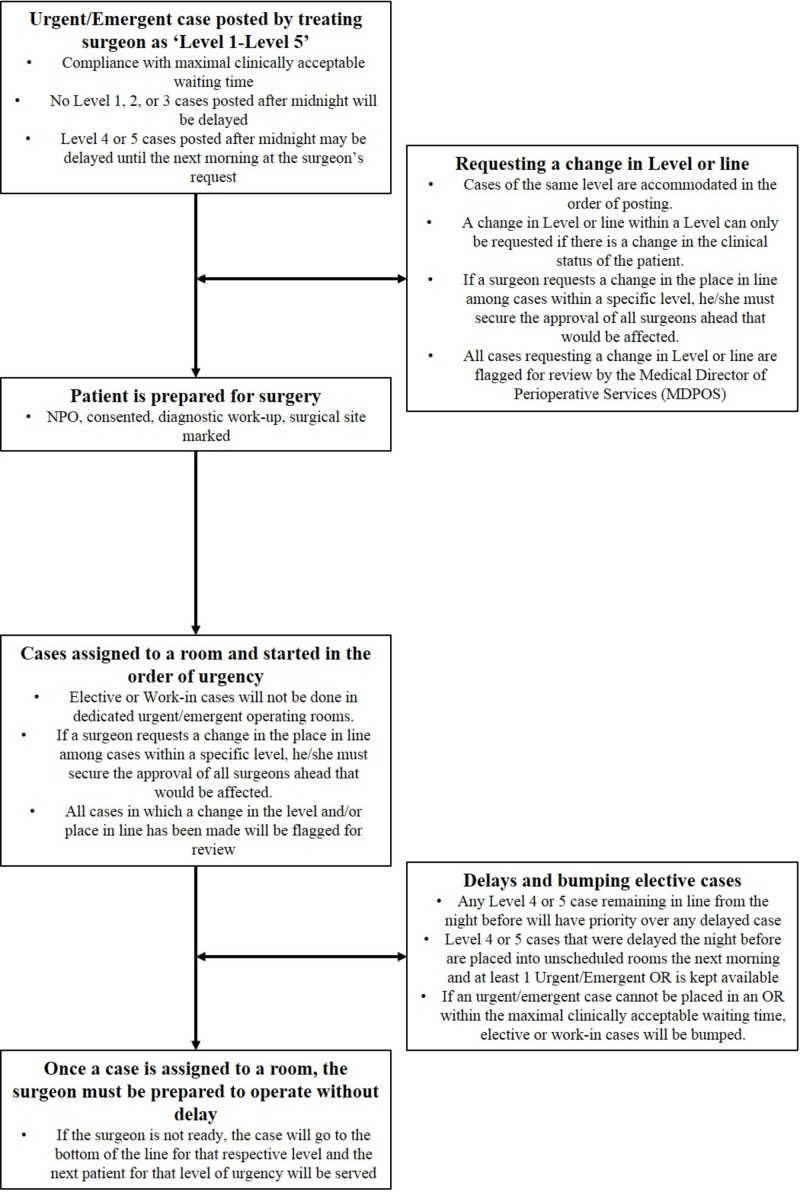
Flow chart for determination of surgical leveling. Flow diagram describing operating room (OR) guidelines for the management of urgent/emergent case flow at a single institution.

Urgent/emergent cases were defined by the institution as patients requiring access to the operating room (OR) within 24 hours of the decision to operate. Clinical need was further classified into five levels, based on the maximum clinically acceptable waiting time between a case being posted and OR access: patient needing surgical intervention within one hour (Level 1), within two hours (Level 2), within six hours (Level 3), within 12 hours (Level 4), and within 24 hours (Level 5). The OR guidelines for the management of urgent/emergent patient flow have been summarized for this institution (Figure 1). An urgent/emergent case is first posted by the treating surgeon, and the patient is prepared for surgery (i.e., NPO, consented, diagnostic workup, surgical site marked). Cases are assigned to an OR and started by level of urgency. Cases within a level are accommodated in the order of posting. The posting surgeon may request a change in the queue within a level or change in the level only if the clinical status of the patient has changed. For urgent/emergent cases that cannot be placed in an OR within the maximal clinically acceptable waiting time, elective cases on the same surgical service are delayed to accommodate the urgent/emergent case. If there are no appropriate elective cases of the same service, then the first available OR of any surgical service is delayed to accommodate the urgent/emergent case. Monthly reports detailing performance regarding maximal clinically acceptable waiting time are distributed to chiefs of each surgical service and reviewed by the institution’s Surgical Executive Committee.

Study design and recorded data

For this retrospective review, all urgent and emergent cases treated at a single institution, the Johns Hopkins Hospital, during a 34-month period (January 1, 2015–October 31, 2017), were identified. All included cases were subject to the Institutional Guidelines for Operative Urgent/Emergent Cases. Operative cases during the study period that were not urgent/emergent were excluded from this study.

Demographic characteristics for urgent cases were compiled by the OR nurse administrator including: the level of urgency (based on institutional guidelines and designated by the treating surgeon), time the case was posted, time the case entered the OR, time the case exited the OR, title of the case, current procedural terminology (CPT) code, surgical specialty, and duration of surgery. The in-room time was calculated as the difference between the time a case exited and entered the operating room. The difference between the time a case was posted and entered the operating room was defined as the waiting period. No protected health information or patient information was collected in this study and Institutional Review Board approval was not required since this was conducted as part of a quality improvement initiative.

Statistical analysis

Continuous demographic data are presented as means with standard deviations. Where applicable, frequencies were compared with Chi-squared tests. A Mann-Whitney U-test was used to compare continuous variables. All analyses were performed in GraphPad Prism 6 (GraphPad Software Inc., La Jolla, California).

## Results

Urgent/emergent cases treated at a large academic Level 1 trauma center over a 34-month period (January 1, 2015–October 31, 2017) were identified, resulting in the inclusion of 11,206 cases (Table 1). Level 2 cases represented the majority of urgent/emergent cases (33%–36%), followed by Level 3 (25%–26%), Level 1 (21%–22%), Level 4 (12%–16%), and Level 5 (2%–4%) (Table 1). Chi-square analysis demonstrated that the proportion of urgent and emergent cases, by level of urgency, did not significantly differ between each year (p > 0.05) resulting in a similar distribution of Level 1 to Level 5 cases.

**Table 1:** Breakdown of number of cases by level, year, and department.

Department	Level 1			Level 2			Level 3			Level 4			Level 5		
	2015	2016	2017^*^	2015	2016	2017	2015	2016	2017	2015	2016	2017	2015	2016	2017
Anesthesiology/Pain Medicine	1	0	2	1	0	1	1	1	2	0	22	27	0	1	2
Cardiothoracic Surgery	86	85	57	74	79	51	27	17	10	16	17	12	11	10	8
Gastroenterology	11	8	6	54	21	22	6	12	22	0	4	15	0	1	2
General Surgery	91	101	63	153	150	122	272	209	152	217	255	253	92	27	20
Interventional Radiology	0	1	0	2	1	0	8	3	0	24	7	1	0	0	0
Neurosurgery	149	150	135	197	166	207	164	128	139	27	41	19	7	6	3
Obstetrics/Gynecology	31	41	36	37	52	45	12	19	8	1	4	0	1	2	0
Ophthalmology	0	2	2	14	11	8	8	15	23	7	11	11	2	2	2
Orthopaedic Surgery	24	27	29	111	90	85	192	217	194	133	187	144	24	15	7
Otolaryngology	105	87	75	78	64	63	51	51	44	18	25	20	7	5	2
Plastic Surgery	17	20	14	53	40	31	83	69	47	7	22	4	6	4	1
Transplant/Abdominal Surgery	52	40	33	287	273	193	6	15	11	0	2	1	4	4	2
Trauma Surgery	171	230	175	222	233	182	120	146	140	2	6	1	1	1	1
Urology	26	30	38	71	55	46	45	38	18	16	12	11	6	2	1
Vascular Surgery	75	42	36	82	57	55	44	36	35	5	3	3	0	0	1
Other	6	5	4	13	13	14	11	17	24	20	22	21	15	8	4
Total	845	869	705	1449	1305	1125	1050	993	869	493	640	543	176	88	56
Proportion of Cases by Level	Level 1			Level 2			Level 3			Level 4			Level 5		
2015	0.21			0.36			0.26			0.12			0.04		
2016	0.22			0.33			0.25			0.16			0.02		
2017	0.21			0.34			0.26			0.16			0.02		

Distribution of urgent/emergent cases

**Figure 2:**
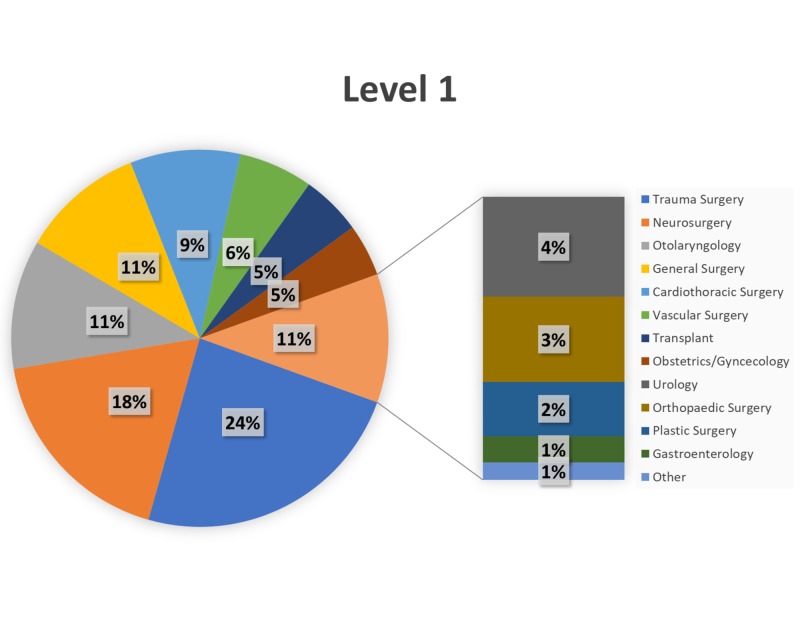
Breakdown of Level 1 cases by service. Breakdown of surgical cases posted as Level 1 by primary service.

**Figure 3:**
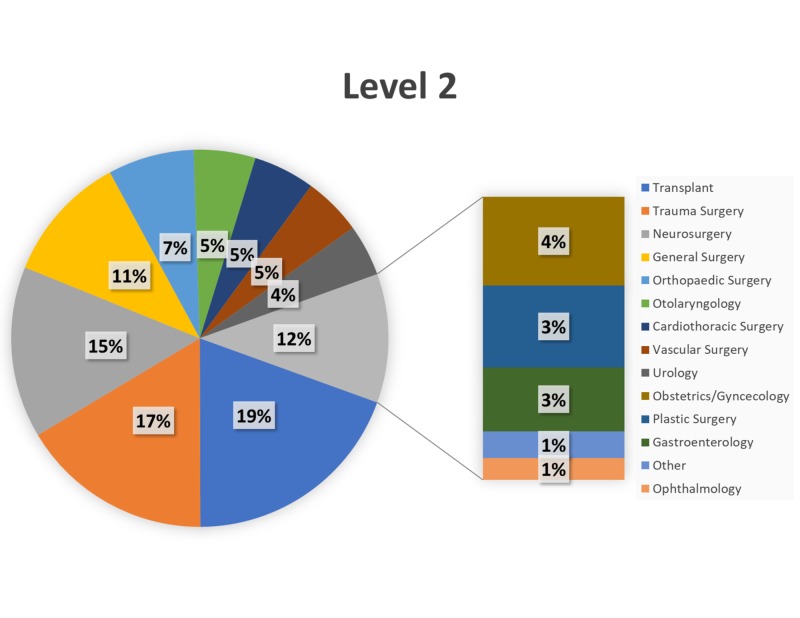
Breakdown of Level 2 cases by service. Breakdown of surgical cases posted as Level 2 by primary service.

**Figure 4:**
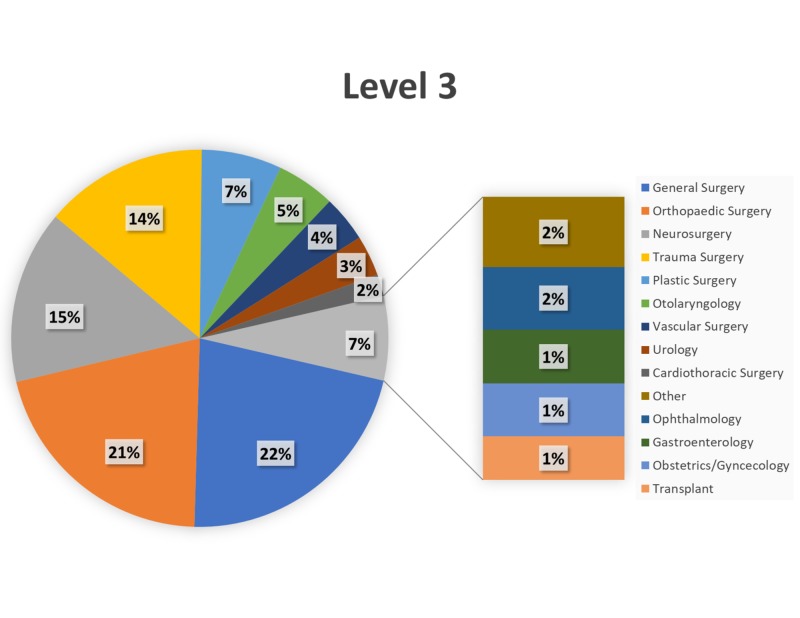
Breakdown of Level 3 cases by service. Breakdown of surgical cases posted as Level 3 by primary service.

**Figure 5:**
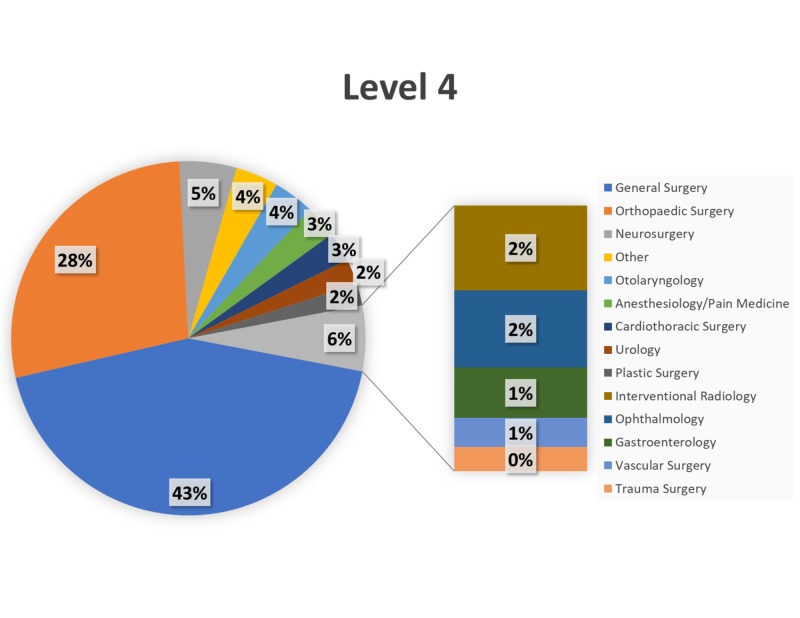
Breakdown of Level 4 cases by service. Breakdown of surgical cases posted as Level 4 by primary service.

**Figure 6:**
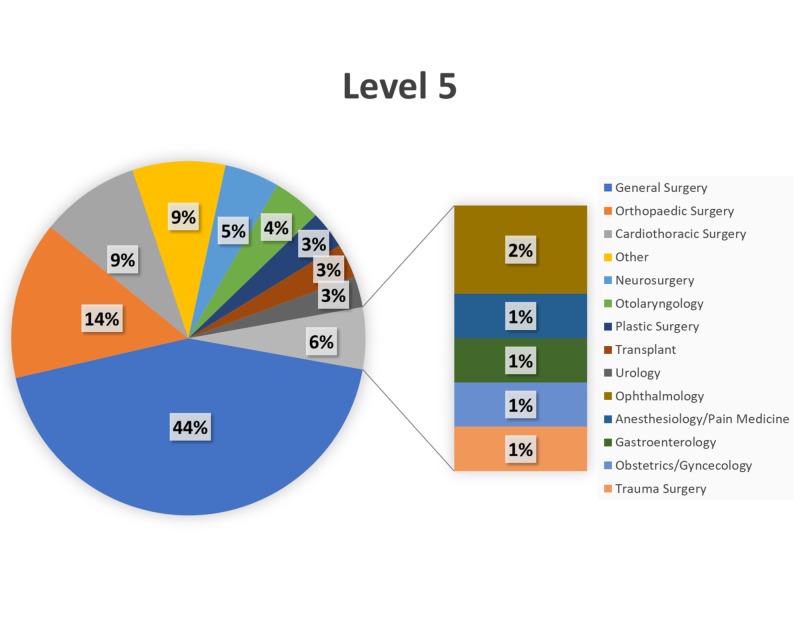
Breakdown of Level 5 cases by service. Breakdown of surgical cases posted as Level 5 by primary service.

Over the 34-month study period (January 1, 2015–October 31, 2017), trauma surgery (24%) and neurosurgery (18%) comprised the most Level 1 cases (Figure 2), with transplant (19%) and trauma surgery (17%) comprising the most Level 2 cases (Figure 3). General surgery and orthopaedic surgery represented the majority of Level 3–Level 5 cases (Figures 4-6).

Among trauma surgery, exploratory laparotomy, appendectomy, and abdominal wound revision/irrigation/exploration/debridement were the most common urgent/emergent cases. Similarly, exploratory laparotomy, appendectomy, and extracorporeal membrane oxygenation (ECMO) cannula placement were the most common general surgery cases. Ventriculoperitoneal (VP) shunt placement, craniotomy for epidural hematoma, and VP shunt revision were the most common neurosurgery cases. Open reduction of the elbow, wound revision/irrigation/exploration/debridement, and open reduction of the femur were the most common orthopaedic surgery cases (Tables 2, 3).

**Table 2:** Most common procedure by department for all leveled cases at the Johns Hopkins Hospital, 2015-2017. PICC: Peripherally inserted central catheter; ECMO: Extracorporeal membrane oxygenation; GI: Gastrointestinal; GJ: Gastrojejunostomy; VP: Ventriculoperitoneal.

Department	Procedure Category	Number	Percentage of All Cases
Anesthesiology/Pain Medicine	PICC Line Insertion	46	75.4%
	Intubation	7	11.5%
	Extubation	3	4.9%
Cardiothoracic Surgery	Wound Revision/Irrigation/Exploration/Debridement	114	20.4%
	ECMO Cannulation	74	13.2%
	Aorta Repair	44	7.9%
	Lung Transplant	44	7.9%
Gastroenterology	Endoscopic Retrograde Cholangiopancreatography	96	52.1%
	Esophagogastroduodenoscopy	55	29.9%
	Endoscopy-Upper GI	10	5.4%
General Surgery	Exploratory Laparotomy	278	12.8%
	Appendectomy	273	12.5%
	ECMO Cannulation	96	4.4%
Interventional Radiology	GJ Tube Placement	27	57.4%
	G/GJ Tube Removal	9	19.1%
	G Tube Placement	4	8.5%
Neurosurgery	VP Shunt Placement	256	16.6%
	Craniotomy for Hematoma	148	9.6%
	VP Shunt Revision	229	9.4%
Obstetrics/Gynecology	Salpingectomy/Salpingo-oophorectomy	54	18.7%
	Uterine Dilatation and Curettage	50	17.3%
	Exploratory Laparoscopy	39	13.5%
Ophthalmology	Globe Repair	42	35.6%
	Blepharoplasty	15	12.7%
	Vitrectomy	15	12.7%
Orthopaedic Surgery	Open Reduction-Elbow	230	15.6%
	Wound Revision/Irrigation/Exploration/Debridement	104	7.0%
	Open Reduction-Femur	72	4.9%
Otolaryngology	Wound Revision/Irrigation/Exploration/Debridement	123	17.7%
	Tracheostomy	81	11.7%
	Bronchoscopy	79	11.4%
Plastic Surgery	Open Reduction-Mandible	54	12.9%
	Wound Revision/Irrigation/ Exploration/Debridement – Chest Wall	38	9.1%
	Wound Revision/Irrigation/Debridement – Hand	25	6.0%
Transplant/Abdominal Surgery	Kidney Transplant	367	39.8%
	Liver Transplant	213	23.1%
	Exploratory Laparotomy	117	12.7%
Trauma Surgery	Exploratory Laparotomy	455	27.9%
	Appendectomy	186	11.4%
	Wound Revision/Irrigation/Exploration/Debridement – Abdomen	164	10.1%
Urology	Ureteral Stent Placement	115	27.7%
	Cystoscopy	45	10.8%
	Wound Revision/ Irrigation/Exploration/Debridement	32	7.7%
Vascular Surgery	Toe Amputation	81	17.1%
	Wound Revision/Irrigation/Exploration/Debridement – Foot	67	14.1%
	Wound Revision/Irrigation/Exploration/Debridement – Leg	29	6.1%
Other	Bone Marrow Biopsy	66	33.0%
	Bronchoscopy	25	12.5%
	Lumbar Puncture	17	8.5%

**Table 3:** Most common procedure type by level and listing department for all leveled cases at the Johns Hopkins Hospital, 2015-2017. PICC: Peripherally inserted central catheter; VP: Ventriculoperitoneal; GJ: Gastrojejunostomy.

Department	Level	Procedure Category	Percentage of Department Cases at this Level
Anesthesiology/Pain Medicine	1	Intubation	100%
	2	Intubation	50.0%
		Intrathecal Pump Revision	50.0%
	3	PICC Line Insertion	75.0%
	4	PICC Line Insertion	85.7%
	5	Three Different Procedures	33.3%
Cardiothoracic Surgery	1	Wound Revision – Thorax	27.2%
	2	Lung Transplant	21.6%
	3	Wound Revision – Thorax	20.4%
	4		33.3%
	5		20.7%
Gastroenterology	1	Endoscopic Retrograde Cholangiopancreatography	56.0%
	2		67.0%
	3	Esophagogastroduodenoscopy	45.0%
	4		63.2%
	5	Three Different Procedures	33.3%
General Surgery	1	Exploratory Laparotomy	41.6%
	2		25.9%
	3	Appendectomy	21.8%
	4		14.5%
	5	Hickman Catheter Placement	7.2%
Interventional Radiology	1	Angiogram	100%
	2	Three Different Procedures	33.3%
	3	GJ Tube Placement	63.6%
	4		62.5%
	5		57.4%
Neurosurgery	1	Craniotomy for Hematoma	19.4%
	2	VP Shunt Placement	15.3%
	3		20.9%
	4		31.0%
	5		25.0%
Obstetrics/Gynecology	1	Exploratory Laparoscopy	20.4%
	2	Salpingectomy	21.6%
		Dilatation and Curettage – Uterus	21.6%
	3		17.9%
	4	Five Different Procedures	20.0%
	5	Wound Revision/Irrigation/Exploration/Debridement – Abdomen	66.7%
Ophthalmology	1	Vitrectomy	50.0%
	2	Globe Repair	45.5%
	3		37.0%
	4		31.0%
	5	Examination Under Anesthesia – Eye	33.3%
Orthopaedic Surgery	1	Fasciotomy	41.3%
	2	Wound Revision/Irrigation/Exploration/Debridement – Leg	11.5%
	3	Open Reduction – Elbow	16.3%
	4		23.1%
	5		15.2%
Otolaryngology	1	Tracheostomy	18.4%
	2	Wound Revision/Irrigation/Exploration/Debridement – Neck	15.6%
	3		16.4%
	4		25.4%
	5	Bronchoscopy	35.7%
Plastic Surgery	1	Wound Revision/Irrigation/Exploration/Debridement – Chest Wall/Breast	17.6%
	2	Open Reduction – Mandible	13.7%
	3		16.8%
	4	Wound Revision/Irrigation/Exploration/Debridement – Leg	18.2%
	5	Cranioplasty	27.3%
Transplant/Abdominal Surgery	1	Exploratory Laparotomy	51.2%
	2	Kidney Transplant	46.6%
	3	Wound Revision/Irrigation/Exploration/Debridement – Abdomen	46.9%
	4	Three Different Procedures	33.3%
	5	Kidney Transplant	40.0%
Trauma Surgery	1	Exploratory Laparotomy	52.1%
	2		22.3%
	3	Appendectomy	25.1%
	4	Appendectomy	22.2%
		Wound Revision/Irrigation/Exploration/Debridement – Foot	22.2%
	5	Three Different Procedures	33.3%
Urology	1	Orchiopexy	26.6%
	2	Ureteral Stent Placement	39.0%
	3	Ureteral Stent Placement	32.7%
	4	Cystoscopy	28.2%
	5	Cystoscopy	22.2%
Vascular Surgery	1	Thrombectomy-Unspecified Vessel	10.5%
	2	Wound Revision/Irrigation/Exploration/Debridement – Foot	23.3%
	3	Amputation – Toe	30.4%
	4	Wound Revision/Irrigation/Exploration/Debridement – Foot	27.3%
	5	Fasciotomy	100%
Other	1	Bronchoscopy	25.0%
	2	Wound Revision/Irrigation/Exploration/Debridement – Chest Wall/Breast	20.0%
	3	Bone Marrow Biopsy	26.9%
	4		50.8%
	5		66.7%

When assessed by level of urgency, exploratory laparotomy was the most common Level 1 procedure and second most common Level 2 procedure. ECMO cannulation and craniotomy for hematoma evacuation were the next most common Level 1 procedures. Kidney transplant, exploratory laparotomy, and abdominal wound revision/irrigation/exploration/debridement were the most common Level 2 procedures. Appendectomy, open reduction of the elbow, leg wound revision/irrigation/exploration/debridement, and esophagogastroduodenoscopy (EGD) were the most common Level 3 and Level 4 cases. The most common Level 5 cases were bone marrow biopsy, bronchoscopy, and Hickman catheter placement (Table 4).

**Table 4:** Most common procedures by level at the Johns Hopkins Hospital, 2015-2017. ECMO: Extracorporeal membrane oxygenation

Level	Procedure Type	Number of Cases	Proportion of All Cases of this Level
1	Exploratory Laparotomy	515	21.3%
	ECMO Cannulation	108	4.5%
	Craniotomy for Hematoma Evacuation	84	3.5%
2	Kidney Transplant	351	9.1%
	Exploratory Laparotomy	326	8.4%
	Wound Revision/Irrigation/Exploration/Debridement – Abdomen	232	6.0%
3	Appendectomy	241	8.3%
	Open Reduction – Elbow	98	3.4%
	Wound Revision/Irrigation/Exploration/Debridement – Leg	96	3.3%
4	Appendectomy	107	6.4%
	Open Reduction – Elbow	107	6.4%
	Esophagogastroduodenoscopy	53	3.2%
5	Bone Marrow Biopsy	18	5.6%
	Bronchoscopy	13	4.1%
	Hickman Catheter Placement	10	3.1%
Any	Exploratory Laparotomy	925	8.3%
	Appendectomy	462	4.1%
	Wound Revision/Irrigation/Exploration/Debridement – Abdomen	396	3.5%

Operating room waiting time

Over the 34-month period, the waiting time between the posting of an urgent/emergent case to when it entered the operating room (post-to-room time) decreased significantly over each year (p < 0.05). The mean post-to-room times from 2015, 2016, and 2017 were 193.40 ± 4.78, 177.20 ± 3.29, and 82.01 ± 2.98, respectively (Table 5). Chi-square previously demonstrated no significant differences with regards to the proportion of each level represented, among all urgent/emergent cases for a given year. Given the consistent distribution of urgency over the three years, it is unlikely that differences in post-to-room time be attributed to greater proportions of Level 1 and Level 2 cases in later years.

**Table 5:** Post-to-room time and case duration of procedures by year.

	2015	2016	2017^*^	Overall	
Post-to-Room Time					
N	4016	3892	3298	11206	
Mean Time (min)	193.40 ± 4.78	177.20 ± 3.29	82.01 ± 2.98	139.40 ± 2.23	
Case Duration					
N	4016	3892	3298	11206	
Mean Time (min)	158.16 ± 1.98	158.07 ± 1.88	164.27 ± 2.90	159.97 ± 1.29	
	Level 1	Level 2	Level 3	Level 4	Level 5
Post-to-Room Time					
N	2419	3879	2912	1676	320
Mean Time (min)	31.22 ± 3.42	148.99 ± 4.40	145.17 ± 2.45	180.44 ± 4.43	573.45 ± 25.88
Case Duration					
N	2419	3879	2912	1676	320
Mean Time (min)	169.46 ± 2.71	200.29 ± 2.57	136.28 ± 2.16	101.58 ± 1.89	125.70 ± 4.85

The mean overall post-to-room time for Level 1 cases, 31.22 ± 3.42 minutes, was significantly below the threshold one hour acceptable waiting time (p < 0.05). When assessed by department, orthopaedic surgery was the only service where the mean post-to-room time exceeded the allowable one hour for Level 1 cases (105.2 ± 69.9 minutes), with a violation frequency present in 9% (7/80) of orthopaedic Level 1 cases. Among Level 2 cases, Chi-square demonstrated that the frequency of post-to-room time violations, exceeding the two-hour allowable period, was only significant for transplant surgery (mean 297.7 ± 35.9 minutes) and cardiothoracic surgery (mean 190 ± 21.5 minutes) (p < 0.05 for both; p > 0.05 for all other departments). All other departments had a mean post-to-room time within the two-hour allowable period, with a frequency of violation that was not significant on Chi-square analysis (p > 0.05 for all). The overall mean post-to-room time was within the allowable time for Level 3–5 cases, as were the mean post-to-room times with respect to department (p > 0.05 for all). The frequency of post-to-room time violations, with respect to level and department, was not significant for any department among Level 3–5 cases (p < 0.05 for all) (Table 5).

## Discussion

Trauma system regionalization of patients with life-threatening emergent and urgent cases to Level 1 trauma centers has demonstrated significant reduction in hospital mortality [10]. Among Level 1 trauma centers, however, there is a lack of established triage protocols to optimize surgical timing. Triage protocols for urgent and emergent operative cases reported in the literature typically focus on general surgery alone—underrepresent the variety of surgical specialties present in a Level 1 trauma center [2,3,7-10]. Additionally, there are few studies demonstrating the implementation and efficacy of such protocols at large-volume centers.

Following a Delphi method of international expert opinions and questionnaires, the World Society for Emergency Surgery (WSES) created a standard triage protocol known as the Timing of Acute Care Surgery classification (TACS) [9]. However, there are no reports describing the implementation or efficacy of this classification system. In a nationwide cohort study of 173,643 general surgery cases, by Mullen et al. [13], laparoscopic cholecystectomy and laparoscopic appendectomy were the most common urgent and emergent surgical cases. However, the study failed to compare the frequency of urgent and emergent cases from other surgical specialties, with respect to institution and study period. In addition, there was a heterogenous population of institutions represented, without stratification for Level 1 trauma centers. In the current study of 11,209 cases, exploratory laparotomy, ECMO cannulation, and craniotomy for epidural hematoma were the most common Level 1 cases, performed by the respective departments of trauma surgery, general surgery, and neurosurgery.

Some authors have suggested the use of dedicated ORs for emergency surgery. However, this is often not feasible or efficient in large volume centers, and has shown mixed results with respect to waiting time [14,15]. In a study implementing dedicated operating rooms for emergency surgery, from a large children’s Level 1 trauma hospital, dedicated ‘add-on’ ORs resulted in decreased elective surgery cancellations but did not significantly impact waiting times for emergency cases designated Priority 1 (≤ 1 hr) or Priority 2 (≤ 4 hr) [15]. It is important to note here that the current institution has two ORs designated as trauma rooms, into which urgent/emergent cases frequently are placed. However, there is no precedent of always having an OR empty and waiting for an emergency as exists at some trauma centers.

Several authors have proposed mathematical algorithms to inform sequencing of urgent/emergent cases [16-18]. In one such model, Dexter et al. [18] summarize three objectives when scheduling emergent operative cases: 1) minimizing wait time, 2) adhering to the posting order, and 3) reflecting medical priority. The protocol implemented at Johns Hopkins Hospital exemplifies these three objectives. Additionally, the mean overall waiting period for an urgent/emergent case entering the OR decreased significantly each year (p < 0.05), resulting in a waiting time that was less than half from 2015 to 2017 (193 vs. 82 minutes). This was accomplished without any significant change in the distribution of urgency between each year. As such, these results suggest that acclimation and multi-departmental practice with an established protocol is necessary in order to match clinically acceptable waiting times for urgent/emergent cases.

Limitations of this study include those inherent to retrospective single-institution studies. The study is also limited in reporting clinical outcomes following implementation of the trauma protocol. We acknowledge that the distribution of urgent/emergent cases may vary from institution to institution, depending on the referral region, relative size of various departments, and other factors. However, to the authors’ knowledge, this is the first study to characterize all urgent and emergent cases at a large academic Level 1 trauma center, outline the specialty and nature of emergent operative cases, and assess the efficacy of the institutional trauma protocol on surgical waiting times. We hope this description of types of urgent/emergent cases and validation of our institution’s protocol for reducing OR waiting time will be helpful to other large-volume Level 1 trauma centers.

## Conclusions

Level 1 trauma centers are capable of caring for every aspect of injury and have 24-hour in-house coverage by general surgeons, with prompt availability of orthopedic surgery, neurosurgery, anesthesiology, emergency medicine, radiology, internal medicine, plastic surgery, oral and maxillofacial surgery, pediatric and critical care. Despite the wide variety of trauma, protocols in the current literature often focus on a single surgical service. To the authors’ knowledge, this is the first study to characterize all urgent and emergent cases at a large academic Level 1 trauma center across all surgical specialties, to outline the specialty and nature of emergent operative cases, and to assess the efficacy of the institutional trauma protocol on surgical waiting times over a 34-month period.
